# Clinical trial protocol of PODRACING: A randomized controlled trial evaluating 3D perfusion zone models for selective arterial clamping during robot‐assisted partial nephrectomy

**DOI:** 10.1002/bco2.70191

**Published:** 2026-03-30

**Authors:** Joris Vangeneugden, Saar Vermijs, Pieter De Backer, Camille Berquin, Nicolaas Lumen, Peter De Kuyper, Yannic Raskin, Bernard Bynens, Frederic Baekelandt, Christophe Ghysel, Frederick Peeren, Ruben De Groote, Geert De Naeyer, Alexandre Mottrie, Stefanie De Buyser, Pieter De Visschere, Charlotte Debbaut, Karel Decaestecker, Charles Van Praet

**Affiliations:** ^1^ Department of Urology Ghent University Hospital, ERN eUROGEN accredited center Ghent Belgium; ^2^ Department of Human Structure and Repair, Faculty of Medicine and Health Sciences Ghent University Ghent Belgium; ^3^ IBiTech‐BioMMedA, Department of Electronics and Information Systems, Faculty of Engineering and Architecture Ghent University Ghent Belgium; ^4^ Department of Urology AZ Maria Middelares Ghent Belgium; ^5^ Department of Urology Genk Belgium; ^6^ Department of Urology AZ Sint‐Jan Brugge Bruges Belgium; ^7^ Department of Urology AZORG Aalst Belgium; ^8^ Biostatistics Unit, Department of Public Health and Primary Care Ghent University Ghent Belgium; ^9^ Department of Radiology and Nuclear Medicine Ghent University Hospital Ghent Belgium

**Keywords:** 3D models, 3D perfusion zone models, partial nephrectomy, RCT, renal cell carcinoma (RCC), robot‐assisted partial nephrectomy (RAPN), segmental clamping, selective clamping, selective renal artery clamping

## Abstract

**Background:**

Performing partial nephrectomy (PN) for suspected renal cell carcinoma (RCC) requires a careful clamping strategy to balance blood loss and postoperative kidney function. For better prediction of individual kidney perfusion, the proposed DIPLANN‐tool visualizes arterial perfusion zones using 3D models from CT scans to support surgeons in planning selective clamping (SC). We hypothesize that using 3D perfusion zone (3DPZ) models allows for more frequent and more accurate SC during robot‐assisted PN (RAPN). Furthermore, it might also benefit patients' health and insight.

**Study Design:**

PODRACING (Planning Operative strategy using a Digital Renal Artery ClampING tool) is a multicentre randomized controlled trial, evaluating the potential benefits of 3DPZ models. Patients will be randomized 1:1 to either the experimental group (DIPLANN‐tool + CT) or the control group (conventional CT alone).

**Endpoints:**

The primary endpoint entails planning and performing, as planned, an SC strategy. Key secondary endpoints include the performed clamping strategy (SC vs main artery clamping [MAC]) and the difference in kidney function at 6 months postoperative. Other secondary and exploratory outcomes include different aspects regarding patients' health, patients' insight and surgeons' benefits.

**Patients and Methods:**

All adult patients with cT1–2 N0 M0 renal cancer planned to undergo RAPN with multiphase CT scan with arterial phase available are eligible for inclusion. Apart from the availability of a 3DPZ model (during a preoperative study visit, while planning the surgical strategy and intraoperatively), study procedures are identical for the study group and control group. For the primary endpoint, the surgeon needs to lock his/her final clamping strategy on the study website. The surgeon can use his/her preferred operating method for RAPN (no limitations in surgical approach or robotic system) and postoperative hospitalization course. Postoperative visits entail visits at 1 month, 3 months, 6 months and 12 months.

**Registration:**

The trial is registered on ClinicalTrials.gov with identifier NCT06536439.^1^ The study was approved by the Belgian Federal Agency for Medicines and Health Products. Eudamed number: CIV‐23‐11‐044854.

## 1 BACKGROUND

For patients diagnosed with localized kidney cancer, two main options exist to surgically remove the kidney tumour.^1^ During radical nephrectomy (RN), the entire kidney is removed. During partial nephrectomy (PN), only the tumour is resected, safeguarding the function of the remaining healthy kidney tissue. The latter procedure is generally preferred; however, not always technically feasible. To resect only the tumour, a balance has to be found in the clamping approach: clamping the blood supply to the kidney assures bloodless tumour resection, yet might compromise the postoperative renal function due to the temporary ischemia. Tumour resection without clamping, on the other hand, might lead to substantial blood loss. That is why ‘selective clamping’ (SC) is proposed. In this approach, only those selective arteries are clamped that perfuse the zone, including the tumour. The main drawback of this strategy is that it is often not clear which arteries should be clamped based on standard preoperative imaging, as misjudgement can lead to a high‐risk surgery with excessive bleeding in case incorrect SC is maintained or futile/risky renal arterial tree dissection is performed. Better prediction of individual kidney perfusion will allow for performing SC more accurately and thus possibly preserve healthier kidney tissue by less extensive ischemia.

With this project, we want to offer the surgeon an easy‐to‐use virtual planning tool that facilitates the decision‐making process regarding the feasibility of PN and the corresponding optimal clamping strategy. This tool uses virtual three‐dimensional (3D) models based on computed tomography (CT) scans to visualize precise information on the different anatomical structures and arterial perfusion zones. This may also improve patients' understanding of their own individual situation.^2^ In a technical validation study by De Backer & Vermijs, the proposed new tool (“Digital PLANning in partial Nephrectomy” = DIPLANN‐tool) demonstrated sufficient accuracy in 92% of patients when planning SC for robot‐assisted PN (RAPN).^3^ However, the tool's clinical added value still needs to be determined.

Our hypothesis entails that the 3D perfusion zone models in combination with conventional CT imaging, in patients diagnosed with localized kidney cancer who are planned to undergo renal cancer surgery, will improve patients' health, broaden patients' insight and facilitate surgical strategy planning, compared to conventional CT imaging alone.

In this report, we provide a detailed and comprehensive description of the PODRACING clinical trial protocol (Version 2.0, dd 20/11/2024), following the SPIRIT reporting guidelines.^4^ This includes an in‐depth overview of the study's overall design, the specific interventions being evaluated, the primary and secondary endpoints, as well as the planned methods for statistical analysis. The trial is registered on ClinicalTrials.gov with identifier NCT06536439.^5^ The study was approved by the Belgian Federal Agency for Medicines and Health Products. Eudamed number: CIV‐23‐11‐044854. A completed SPIRIT checklist is available as an additional file (Supporting Information).

## 2 STUDY DESIGN

PODRACING (Planning Operative strategy using a Digital Renal Artery ClampING tool) is a confirmatory, multicentric, unblinded, randomized, controlled, pivotal trial using parallel group assignment and stratified randomization.

Patients will be randomized according to a 1:1 allocation ratio to either the experimental group (the DIPLANN‐tool in combination with conventional CT imaging) or the control group (conventional CT imaging alone), using permuted block randomization with blocks of varying size.

Randomization will be stratified by the following variables:

- Whether SC is deemed possible either according to the DIPLANN‐tool in combination with conventional CT imaging and/or according to conventional CT imaging only, as assessed by an independent surgeon (between inclusion and randomization) who will not be involved in the RAPN surgical procedure (yes vs no), see 5.2 ‘study procedures’ for more details.
- Hospital where surgery is performed (Ghent University Hospital vs AZ Maria Middelares Ghent vs AZORG Hospital Aalst vs AZ Sint‐Jan Bruges vs ZOL Genk).
- PADUA classification (low (<8) and intermediate (8–9) vs high‐risk (>9)). In case of multiple masses, the mass with the highest individual PADUA classification will be used.

A static randomization list was developed by an independent statistician and uploaded in the randomization module of REDCap to allow for automated randomization.

The study will be rolled out in five high‐volume hospitals in Belgium (Ghent University Hospital, AZ Maria Middelares Ghent, AZORG Hospital Aalst, AZ Sint‐Jan Bruges and ZOL Genk), including surgeons with different ranges of experience in RAPN and in 3D modelling. We aim to randomize 235 patients diagnosed with localized kidney cancer who are planned to undergo renal cancer surgery.

## 3 ENDPOINTS

An overview of the primary endpoint, as well as key secondary, safety and exploratory endpoints, is provided in Table 1.

**TABLE 1:** Overview of primary and secondary endpoints.

**Primary Endpoint**
Planning and performing as planned a SC strategy (considered by the surgeon at the end of surgery, objectified pre‐ and postoperatively through online assessment and controlled through video‐analysis by trained reviewers using predefined criteria)*
**Secondary Endpoints**
*Key Secondary Endpoints*
Change in eGFR at 6 months after surgery compared to eGFR preoperatively
Clamping strategy performed (SC or MAC)
*Secondary Endpoints*
Time to dissect hilum (minutes) as analysed on postoperative surgical video analysis
Conversion from SC to MAC
*Safety Endpoints*
Intra‐operative and 90‐day postoperative complications
Estimated blood loss during surgery (mL)
Concordance between the 3D perfusion model and peroperative kidney surface perfusion as visualized by indocyanine green (ICG) as estimated by postoperative surgical video analysis
*Exploratory Endpoints*
Patients' health
eGFR (CKD‐EPI) < 45 ml/min at 6 months after surgery
Change in eGFR (CKD‐EPI) after surgery at other time points up to 1 year (POD1, last measurement before discharge, postoperatively 1 month, 3 months, 12 months) compared to eGFR preoperatively
Ischemia time during surgery (seconds)
Positive surgical margin (PSM)
Robotic console time of surgery (minutes)
Length of hospital stay after surgery (days)
2 Patients’ insight
Quality of life (according to EORTC QLQ‐C30) up until 1 year after surgery (preoperatively, 3 months, 6 months, 12 months
Preoperative anxiety for surgery questionnaire (Hospital Anxiety and Depression Scale (HADS) + Symptom Distress Thermometer (SDT))
Preoperative patient knowledge questionnaire
3 Surgeon's decision
Time to dissect kidney (minutes)
Superselective clamping (yes/no)
% parenchyma rendered ischemic
Conversion from PN to RN
Time to prepare for surgery (minutes)

* For the primary objective, SC needs to be deemed possible either according to the DIPLANN‐tool in combination with conventional CT imaging or according to conventional CT imaging only, as assessed by an independent surgeon (between inclusion and randomization) who will not be involved in the RAPN surgical procedure, in order to be included in the analysis set. On the DIPLANN tool, SC is deemed feasible if ≥ 90% tumour ischemia and ≤ 70% renal parenchyma ischemia can be achieved. If these criteria are met, but it is technically or anatomically not feasible according to the independent surgeon to perform SC, he can deviate from these criteria and thus claim SC is not deemed possible. As a secondary objective, this endpoint will be analysed in the total study population.

The primary endpoint entails planning and performing, as planned, an SC strategy (considered by the surgeon at the end of surgery, objectified pre‐ and postoperatively through online assessment and controlled through video‐analysis by trained reviewers using predefined criteria). The surgical procedure of a patient is deemed to have a favourable outcome if an SC strategy is planned and performed as planned. A surgical procedure of a patient is considered to have an unfavourable outcome if main artery clamping (MAC) is planned, or if SC is planned, but cannot be performed as planned (other or adjusted SC is performed or MAC is performed). The primary endpoint will only be analysed in the subgroup of patients in whom SC is deemed possible (stratification factor, see ‘study procedures’). The secondary endpoints will be analysed for all patients.

## 4 ELIGIBILITY CRITERIA

Inclusion and exclusion criteria are summarized in Table 2. We expect SC to be deemed possible (either according to the DIPLANN‐tool in combination with conventional CT imaging or according to conventional CT imaging only) in 202 of them (see sample size calculation under *Methods*) to assess our primary objective. No specific number of randomized patients per hospital is required.

**TABLE 2:** Inclusion and exclusion criteria for participation in the PODRACING trial.

**Inclusion criteria**
Aged 18 years or above
cT1–2 N0 M0 renal cancer
Planned to undergo RAPN
Multiphase CT scan with arterial phase available
Voluntary given and written informed consent
Proficiency in at least one of the study languages: Dutch, English, French
**Exclusion criteria**
> 3 ipsilateral renal masses
Women who are pregnant or breastfeeding
Previous renal surgery that is expected to complicate renal cancer surgery
cT ≥ 3
Planned off‐clamp resection
Cognitive disorder which impedes with completing study questionnaires

## 5 METHODS

### 5.1 Interventions

#### 5.1.1 Description of the medical device

**FIGURE 1:**
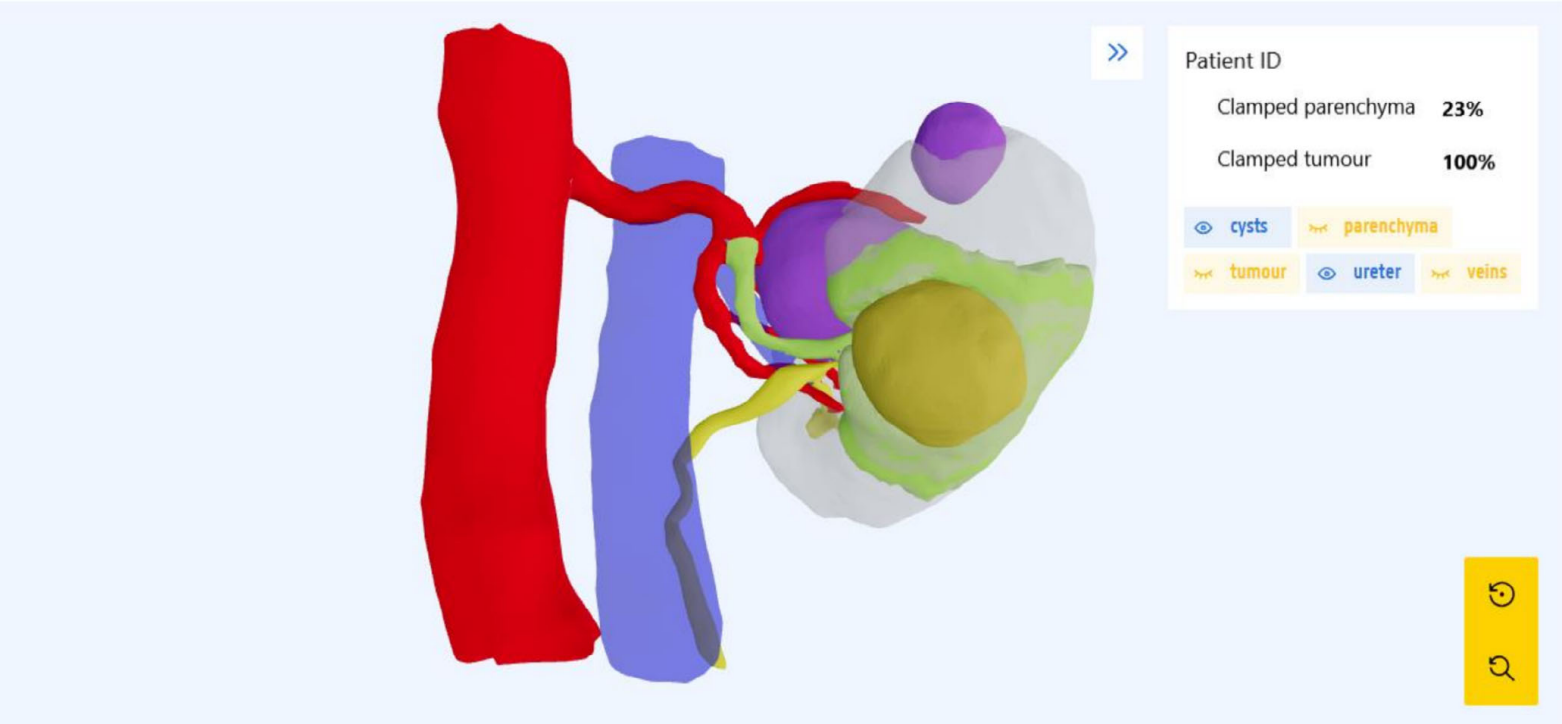
Visual from the DIPLANN website (IBiTech‐BioMMedA, Ghent University, Ghent, Belgium). Arteries in red, veins in blue, parenchyma in grey, cysts in purple, tumour in orange, ureter and pyelocaliceal system in yellow, perfusion zone in green. The 3D perfusion zone model can be rotated, and structures can be toggled on/off, and put on transparent. Arteries can be explored to see which artery perfuses which zone of the kidney. The percentage of estimated clamped parenchyma and clamped tumour can be noted in the top right. The surgeon can use this information to plan his surgical strategy. The model can be visualized on an adjacent PC, smartphone or tablet or can be visualized through the TilePro screen of the DaVinci surgical robot (Intuitive Surgical, Sunnyvale, CA, USA).

The medical device (DIPLANN‐tool) used in the study group is a software (name: DIPLANN – version: 2.0) that calculates the perfusion zones in the kidney and visualizes this information on a 3D model rendered in a web‐based environment (Figure 1). This information can be used by the surgeon to check for each renal arterial branch, which part of the tissue will become ischemic should it be clamped. Using this information, the surgeon can plan a surgical strategy (clamping strategy) and explain it visually to the patient.

3D models will be created from CT images using the 3D modelling platform from an external, commercial company (MEDANNOT s.r.o., Spania Dolina, Slovakia). On this platform, an automated AI‐generated first segmentation is made (using our own previous 3D data^3^). Afterwards, this segmentation is further handled by the study team and an experienced uroradiologist to ensure correct segmentation and add final detail. Afterwards, on this 3D model, a perfusion zone algorithm is used, developed by Ghent University (research group IBiTech – BioMMedA, Department of Electronics and Information Systems, Faculty of Engineering and Architecture).

### 5.2 Study procedures

**FIGURE 2:**
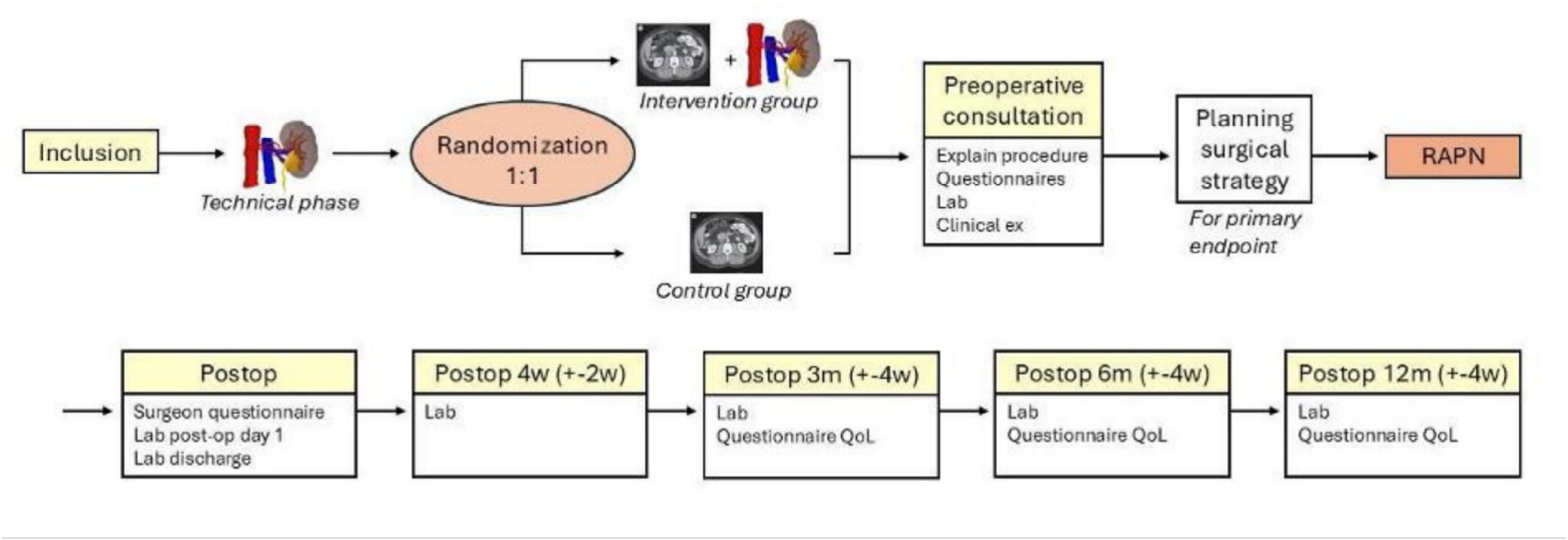
Schematic presentation of the PODRACING workflow.

An overview of the study flow is presented in Table 3 and Figure 2.

**TABLE 3:** Overview of all study‐specific procedures, following SPIRIT guidelines.^4^

TIMEPOINT	STUDY PERIOD											
	Enrolment		Allocation	Post‐allocation								Close‐out
	Screening (visit)	Technical	Randomization	Pre‐op (visit)	Day before operation (or earlier after pre‐op visit)	Day of operation	Post‐op day 1	Discharge *	1 m (visit) **	3 m (visit) ***	6 m (visit) ***	12 m (visit) ***
**ENROLMENT:**												
**Eligibility screen**	x											
**Informed consent**	x											
**Sending CT images to Central Imaging Analysis Centre for design of 3D models and perfusion models (including validation by uro‐radiologist)**		x										
**Possibility of selective clamping assessed by independent surgeon**		x										
**Allocation**			x									
**INTERVENTIONS:**												
***3D perfusion model + CT available for patient counselling, preoperative planning and perioperative guidance***			x									
***Only CT available***			x									
**ASSESSMENTS:**												
**Preoperative study visit explaining the procedure using either CT scan or 3D model**				x								
**Physical examination including height, weight, abdominal examination**				x								
**Blood examination including Hb, creatinine, eGFR**				x			x					
**Blood examination including creatinine, eGFR**								x	x	x	x	x
**Patient questionnaire regarding knowledge**				x								
**Hospital Anxiety and Depression Scale (HADS) + Symptom Distress Thermometer (SDT)**				x								
**EORTC QLQ‐C30 questionnaire**				x						x	x	x
**Online assessment by surgeon regarding selective arterial clamping information**					x							
**RAPN surgical procedure**						x						
**Postoperative questionnaire for the surgeon with surgical details and information on arterial clamping**						x						
**Histopathological examination of renal tumour**						x						
**Follow‐up consultations and monitoring of complications**									x	x	x	x

* Last measurement before discharge.

** Time window of 2 weeks before and 2 weeks after.

*** Time window of 4 weeks before and 4 weeks after.

After checking eligibility, informed consent is signed, and anonymized CT images are sent to a central imaging analysis team for design of the 3D models (using the MEDANNOT 3D segmenting platform) and perfusion zone models (using the DIPLANN tool by IBiTech‐BioMMedA, Ghent University). The 3D models and the perfusion zone models will be manufactured centrally, under the supervision of an expert uroradiologist. A central independent surgeon will assess if SC is deemed possible on CT and/or the DIPLANN tool (for the primary objective and for stratification), first on CT only, afterwards on the DIPLANN tool. On the DIPLANN tool, SC is deemed feasible by the independent surgeon if ≥ 90% tumour ischemia and ≤ 70% renal parenchyma ischemia can be achieved. If these criteria are met, but it is technically or anatomically not feasible according to the independent surgeon to perform SC, he can deviate from these criteria and thus claim SC is not deemed possible. These feasibility thresholds were selected based on expert consensus and prior technical implementation of the perfusion zone models and represent a pragmatic balance between achieving near‐complete tumour ischemia for oncological safety and limiting ischemic exposure of healthy renal parenchyma. After this, randomization will take place and the local team of the respective study site will be notified to which group the patient is assigned. If randomized in the study group, the 3D perfusion zone model will be accessible on the DIPLANN website for the treating surgeon at the local study site. The surgeon can now explain the procedure to the patient using the 3D model. If the patient is randomized in the control group, there will be no 3D model available on the DIPLANN website for the surgeon, and he/she continues only with the CT scans.

Apart from the availability of a 3D perfusion zone model, study procedures are identical for the study group and control group.

Next to explaining the procedure with or without the study model, a preoperative visit consists of checking for preoperative kidney function and haemoglobin, an abdominal examination and completing quality of life, knowledge and anxiety questionnaires.

For the primary endpoint, the surgeon needs to lock his/her final clamping strategy on the DIPLANN website (with or without the use of a 3D model) and time his/her planning process with a built‐in timer system. After locking in the clamping strategy, he/she can now proceed with the RAPN procedure (with or without the peri‐operative use of a 3D model). The surgeon can use his/her preferred operating method for RAPN (no limitations in surgical approach or robotic system). Important during the surgery is the use of indocyanine green (ICG) after clamping the artery (for a secondary validation endpoint) and recording the surgery, to objectively note surgery details (e.g. hilar dissection time). Postoperative hospitalization course is fully decided by the surgeon. Blood examination results of the first postoperative day and last before discharge are collected.

Postoperative visits entail visits at 1 month, 3 months, 6 months and 12 months for adverse event monitoring, kidney function follow‐up and/or follow‐up quality of life questionnaires. Postoperative imaging happens outside of the study and at the treating physician's pace of choice (usually according to current EAU guidelines for RCC follow‐up.^1^

### 5.3 Sample size determination

The study is powered for its primary objective, which targets patients in whom SC is deemed possible either according to the DIPLANN‐tool in combination with conventional CT imaging or according to conventional CT imaging only, as assessed by an independent surgeon between inclusion and randomization.

A sample size of 196 patients in whom SC is deemed possible is needed to achieve at least 80% power to detect an absolute risk difference of 15.6% in favourable outcome (planning and performing as planned a SC strategy) between the DIPLANN‐tool in combination with conventional CT imaging and conventional CT imaging alone, using an exact binomial test at the two‐sided 5% significance level when the design is balanced (1:1 allocation ratio) and the true rate of favourable outcome is 73.4% with conventional CT imaging alone and 89.0% with the DIPLANN‐tool in combination with conventional CT imaging.

The sample size was increased to 198 patients to allow for a 1% drop‐out. The sample size was further increased to 202 patients in whom SC is deemed possible (101 in each group), to foresee an interim analysis for efficacy when 50% of the primary endpoints are measured, using a group sequential design with O'Brien Fleming alpha spending function.

When SC is deemed possible in 86% of patients, the total sample size of patients diagnosed with localized kidney cancer who are planned to undergo renal cancer surgery will be 235. The assumed rate of SC performed as predicted, with a standard of care of 73.4%, is based on other studies.^6^, ^7^

Recruitment will continue until 202 patients in whom SC is deemed possible are randomized.

### 5.4 Methods of data collection

Data collection will comply with GDPR (EU 2016/679). Source data will be recorded at the subject's visit, with protocol‐required data entered into eCRFs in REDCap, a secure web‐based system licensed to HIRUZ.

Authorized study staff will enter and correct data in accordance with ISO 14155. eCRFs will be signed and verified for accuracy and completeness by the principal investigator or a co‐investigator, and reviewed by trained monitors. Data cleaning will be performed by the sponsor. Patient questionnaires will be entered directly as source data, and essential documents will be retained for at least 10 years in line with regulatory requirements.

### 5.5 Recruitment and consent

Recruitment will take place at the urology departments of the different participating hospitals by a participating clinical investigator. This can only take place after radiographic confirmation of a renal lesion on a multiphase CT scan.

Prior to entry in the study, the investigator will explain to potential subjects the study and the implications of participation. Subjects will be informed that their participation is voluntary and that they may withdraw consent to participate at any time. Participating subjects will be told that their records may be accessed by competent authorities and by authorized persons without violating the confidentiality of the subject, to the extent permitted by the applicable law(s) and/or regulations. By signing the Informed Consent Form (ICF), the subjects are authorizing such access.

### 5.6 Safety reporting

This study will be conducted in accordance with the protocol, ISO 14155 (Good Clinical Practice), and applicable national and European regulations, including GDPR (EU 2016/679), the EU Medical Device Regulation (MDR 2017/745) and relevant Belgian legislation.

Adverse events (AEs) will be reported from the first use of the medical device until the last study‐related activity. Events occurring between informed consent and first device use will be recorded as medical history. All (S)AEs will be documented in the patient file and eCRF, except hospitalizations for social reasons or unchanged pre‐existing conditions. All serious adverse events (SAEs) and device deficiencies will be reported to the sponsor/national coordinator and monitor within three calendar days using the appropriate forms, with follow‐up information provided as required.

### 5.7 Authorization

This study obtained approval from the Regulatory Authorities (RA) prior to the start of the study. Substantial protocol modifications will be submitted to the RA during the course of the study according to the requirements and within the timelines as defined by the national law. Substantial modifications will only be implemented after approval of the RA. The submission package will include a cover letter including a rationale or justification of the changes (point by point), a list of documents submitted, an application form, amended documents in track change and clean version and any other documents that may be relevant for the assessment of the modification as applicable.

### 5.8 Monitoring

Regular monitoring will be performed by HIRUZ CTU according to ICH GCP and ISO 14155. Data will be evaluated for compliance with the protocol and accuracy in relation to source documents. Following written standard operating procedures, the monitors will verify that the clinical investigation is conducted and data are generated, documented and reported in compliance with the protocol, ISO 14155 and the applicable regulatory requirements. An initiation visit, routine monitoring visits and a final visit after the last patient had finished the study. The frequency, extent and nature of the monitoring will depend on the risk assessment of the study.

### 5.9 Analysis plan

Statistical analysis will be performed by the investigating team, with the support of the Biostatistics Unit of Ghent University.

#### 5.9.1 Summary of baseline data

Descriptive statistical analyses will be performed for patient characteristics. Tests of statistical significance will not be undertaken for baseline characteristics; rather, the clinical importance of any imbalance will be noted.

#### 5.9.2 Primary estimand

Our primary estimand is the absolute difference in proportion of favourable outcome (planning and performing as planned a SC strategy) between the DIPLANN‐tool in combination with conventional CT imaging and conventional CT imaging alone, in patients diagnosed with localized kidney cancer who are planned to undergo renal cancer surgery and *in whom SC is deemed possible* either according to the DIPLANN‐tool in combination with conventional CT imaging or according to conventional CT imaging only. Intercurrent events such as cancellation or postponement of the surgical procedure of a randomized patient outside of the study time limit will be dealt with using a composite strategy: the surgical procedure of a patient with such an intercurrent event is considered to have an unfavourable outcome. Given that we expect these intercurrent events to occur equally in both arms, this will lead to a conservative estimate of the risk difference. Intercurrent events such as major protocol deviations and a switch in the medical device used for planning the surgical procedure will be dealt with using the treatment policy strategy to determine the effect including all outcomes regardless of these intercurrent events occurring (similar to the intention to treat principle).

#### 5.9.3 Primary estimator (primary analysis) and estimate

We will apply a statistically reliable method of covariate adjustment to estimate the marginal risk difference as an unconditional treatment effect. This standardized estimator is recommended in the U.S. Food and Drug Administration Guidance on covariate adjustment (FDA 2023).

A binary logistic regression model will be fitted with maximum likelihood on each arm separately to regress the primary endpoint on hospital and the highest individual PADUA classification (low (<8), intermediate (8–9) and high‐risk (>9)). The model will include an intercept term. The probability of a favourable primary outcome will be predicted for all subjects twice: once based on the model fitted on the arm assigned to the DIPLANN‐tool in combination with conventional CT imaging, and once based on the model fitted on the arm with conventional CT imaging alone. Hence, there are two scenarios under which the counterfactual probabilities are calculated: 1) everyone is assigned to DIPLANN‐tool in combination with conventional CT imaging, 2) everyone is assigned to conventional CT imaging alone.

The average responses under both scenarios are estimated by averaging the respective counterfactual probabilities across all subjects. These estimates of average response rates in the two treatment groups are used to estimate the unconditional absolute risk difference.

A confidence interval (CI) around the marginal risk difference will be computed using the non‐parametric BCa bootstrap method (Efrond and Tibshirani 1994).

This analysis will include all randomized patients in whom SC was deemed possible, either according to the DIPLANN‐tool in combination with conventional CT imaging or according to conventional CT imaging only.

#### 5.9.4 Interim analysis for efficacy

When 50% of the primary endpoints are measured, a non‐binding interim analysis for efficacy for the primary objective and primary estimand will be performed at the 0.88% significance level (z = 2.37, based on O'Brien Fleming group sequential boundaries).

The final analysis of the primary endpoint will be performed at the 4.67% significance level (z = 1.68).

#### 5.9.5 Subgroup analyses

To explore the heterogeneity of the primary estimand, subgroup analyses will be performed for the following subgroups:

- Hospital where surgery is performed (Ghent University Hospital vs AZ Maria Middelares Hospital vs other hospitals).
- Kidney function (eGFR < 30, [30–45[, [45–60[, ≥ 60).
- Highest individual PADUA classification (low (<8), intermediate (8–9) and high‐risk (>9)).

#### 5.9.6 Key secondary estimand 1 and estimator

One of our key secondary estimands is very similar to the primary estimand, but targets a different patient population, namely all patients diagnosed with localized kidney cancer who are planned to undergo renal cancer surgery (regardless of whether SC is deemed possible). The analysis will be similar to the analysis for the primary estimand, but will include all randomized patients and will be adjusted for whether SC was deemed possible by the independent surgeon (as a main effect).

#### 5.9.7 Key secondary estimand 2 and estimator

Another key secondary estimand is the difference in mean change from baseline (preoperative) in eGFR at 6 months postoperatively, between the DIPLANN‐tool in combination with conventional CT imaging and conventional CT imaging alone, in all patients diagnosed with localized kidney cancer who are planned to undergo renal cancer surgery.

Because eGFR is measured repeatedly (preoperatively, POD1, last measurement before discharge, postoperatively 1 month, 3 months, 6 months, 12 months), we will fit a linear mixed model for eGFR with a random intercept for patient, including time, arm, a two‐way interaction between time and arm and the stratification factors: whether SC is deemed possible, hospital and highest individual PADUA classification in the fixed effects part of the model.

#### 5.9.8 Key secondary estimand 3 and estimator

A final key secondary estimand is the absolute difference in proportion of SC strategy performed (favourable outcome SC versus unfavourable outcome MAC) between the DIPLANN‐tool in combination with conventional CT imaging and conventional CT imaging alone, in all patients diagnosed with localized kidney cancer who are planned to undergo renal cancer surgery. The same intercurrent events can be expected as for the primary estimand, and these will also be dealt with using the same strategies.

#### 5.9.9 Other analyses

Endpoints that are measured repeatedly will be analysed using generalized linear mixed models with a random intercept for patient, including time, arm, a two‐way interaction between time and arm and the stratification factors: whether SC is deemed possible, hospital, and the highest individual PADUA classification in the fixed effects part of the model.

Continuous endpoints measured at one point in time will be analysed using a linear regression model including arm and the stratification factors: whether SC is deemed possible, hospital and the highest individual PADUA classification. Log‐normally distributed endpoints will be log‐transformed before analysis.

Binary endpoints measured at one point in time will be analysed using a binary logistic regression model (with logit link function), including arm and the stratification factors: whether SC is deemed possible, hospital and the highest individual PADUA classification.

#### 5.9.10 Missing data

Given the pragmatic design of the study, a few missing data points can be expected. Furthermore, we do not expect that the probability of our endpoint being missing depends on the unobserved value of the endpoint or on the observed values of other recorded values. Therefore, under the assumption of data being missing completely at random (MCAR), a complete‐case analysis will still provide a valid inference.

Endpoints that are measured repeatedly will be analysed using longitudinal likelihood‐based analyses that will make use of all observed data and will provide valid inferences when outcome data is missing at random (MAR).

#### 5.9.11 Significance level and confidence intervals

The nominal type I error rate will be controlled at 5%. The hierarchy of endpoint families will be respected. Analysis of secondary endpoints will only be interpreted after demonstration of success on the primary estimand.

The interim analysis for the primary estimand will be performed at the 0.88% significance level and the final analysis at the 4.67% significance level (based on O'Brien Fleming group sequential boundaries).

Bonferroni correction for multiple testing due to multiple key secondary estimands will be applied, setting the significance level at 1.67%.

Subgroup analyses and analyses of other endpoints will be considered as exploratory and hypothesis‐generating.

Any deviation(s) from the original statistical analysis plan will be recorded as a note to file and reported in the final clinical investigation report and publication.

## 6 DISCUSSION

In an era where RAPN has emerged as the preferred approach for the treatment of small renal masses, this shift has been accompanied by the progressive integration of advanced technologies aimed at optimizing surgical outcomes. One such innovation is the use of patient‐specific 3D anatomical kidney models. These models have the potential to enhance intraoperative precision by supporting meticulous hilar dissection and improving tumour localization. Additionally, perfusion zone mapping can be superimposed onto the 3D model to delineate vascular territories, thereby guiding selective arterial clamping and contributing to the preservation of uninvolved renal parenchyma.

These models are inherently intuitive and have been shown to improve tumour complexity understanding, facilitate a more selective clamping approach, incur less complications and sometimes even better postoperative kidney function outcomes.^8^, ^9^ As most literature is based on purely retrospective data, we aim to further substantiate these findings in a prospective, randomized, multicentre trial.

In the present trial, renal function was not designated as the primary outcome, as this is influenced by a multitude of factors beyond the mere availability of a 3D model. One such factor could be the adoption of more selective clamping strategies. However, to date, several studies have failed to demonstrate a clear benefit of SC or off‐clamp resection on postoperative renal function.^10^, ^11^, ^12^ It is important to note that many of these studies included patients with preserved baseline renal function, often involved cross‐over from SC to MAC, and did not incorporate the use of 3D models—particularly those incorporating perfusion zone information. Nonetheless, renal function remains a key secondary outcome in this trial and will be analysed in relation to surgical technique, clamping strategy and other perioperative variables. Future research should evaluate the use of 3D models in conjunction with SC in patient populations with impaired renal function (patients with a solitary kidney or chronic kidney disease) or complex renal tumours.

With this RCT, we investigate whether the use of a 3D perfusion zone model increases the successful performance of SC during RAPN. Alongside this, PODRACING includes a comprehensive assessment of patients' health outcomes, patients' insight and surgeons' benefits.

## AUTHOR CONTRIBUTIONS

*Conceptualization*: Joris Vangeneugden, Saar Vermijs, Pieter De Backer, Charlotte Debbaut, Karel Decaestecker, Charles Van Praet. *Methodology*: Joris Vangeneugden, Saar Vermijs, Charlotte Debbaut, Karel Decaestecker, Charles Van Praet. *Software*: Saar Vermijs, Charlotte Debbaut. *Data curation*: Joris Vangeneugden, Saar Vermijs. *Investigation*: Joris Vangeneugden, Pieter De Backer, Camille Berquin, Nicolaas Lumen, Peter De Kuyper, Yannic Raskin, Bernard Bynens, Frederic Baekelandt, Christophe Ghysel, Frederick Peeren, Ruben De Groote, Geert De Naeyer, Alexandre Mottrie, Karel Decaestecker, Charles Van Praet. *Validation*: Joris Vangeneugden, Saar Vermijs, Pieter De Backer, Charlotte Debbaut, Karel Decaestecker, Charles Van Praet. *Formal analysis*: Joris Vangeneugden, Stafanie De Buyser, Charles Van Praet. *Supervision*: Charlotte Debbaut, Karel Decaestecker, Charles Van Praet. *Funding acquisition*: Charlotte Debbaut, Karel Decaestecker, Charles Van Praet. *Visualization*: Joris Vangeneugden, Saar Vermijs, Pieter De Backer, Pieter De Visschere, Charlotte Debbaut. *Project administration*: Joris Vangeneugden, Charles Van Praet. *Resources*: Bernard Bynens, Christophe Ghysel, Alexandre Mottrie, Pieter De Visschere, Charlotte Debbaut, Karel Decaestecker, Charles Van Praet. *Writing—original draft*: Joris Vangeneugden. *Writing—review and editing*: Saar Vermijs, Pieter De Backer, Camille Berquin, Nicolaas Lumen, Peter De Kuyper, Yannic Raskin, Bernard Bynens, Frederic Baekelandt, Christophe Ghysel, Frederick Peeren, Ruben De Groote, Geert De Naeyer, Alexandre Mottrie, Stefanie De Buyser, Pieter De Visschere, Charlotte Debbaut, Karel Decaestecker, Charles Van Praet.

## CONFLICT OF INTEREST STATEMENT

There are no disclosures other than the funding already disclosed.

## Supporting information




**Data S1.** Supporting Information.
