# Correction to ‘Clinical trial protocol of PODRACING: A randomized controlled trial evaluating 3D perfusion zone models for selective arterial clamping during robot‐assisted partial nephrectomy’

**DOI:** 10.1002/bco2.70254

**Published:** 2026-07-21

Vangeneugden J , Vermijs S , De Backer P , Berquin C , Lumen N , De Kuyper P , et al. Clinical trial protocol of PODRACING: A randomized controlled trial evaluating 3D perfusion zone models for selective arterial clamping during robot‐assisted partial nephrectomy. BJUI Compass. 2026;7(4):e70191. 10.1002/bco2.70191 42022229 PMC13098362

We would like to disclose a competing interest that was inadvertently omitted from the published Conflict of Interest Statement. Since March 2024, author Pieter De Backer has been a co‐founder and shareholder of MEDANNOT s.r.o. (Spania Dolina, Slovakia). As described in the Methods, the trial uses the Medannot 3D segmentation platform to create the 3D models, so the financial interest in this company is relevant to the study and should have been declared.

This omission was not intentional. The same oversight affected the Funding section, in which PDB's VLAIO Baekeland mandate (HBC.2020.2252), active at the time, was also not listed.

The revised conflict of interest statement and added funding information are as follows:

Conflict of Interest (amended): “Pieter De Backer (P.D.B.) has been a co‐founder and shareholder of MEDANNOT s.r.o. (Spania Dolina, Slovakia) since March 2024. The Medannot 3D modelling platform was used in this study. This constitutes a competing interest that was not declared in the original publication. The competing‐interest declarations of the remaining authors are unchanged.”

Funding (addition): “Pieter De Backer was supported by a VLAIO Baekeland mandate (HBC.2020.2252).”

We apologise for these errors.

